# Natural variation in the promoter of *OsPDX1.2* enhances vitamin B6 accumulation and cold tolerance in rice

**DOI:** 10.1111/jipb.70322

**Published:** 2026-06-15

**Authors:** Bi Wang, Qiu Yi Wang, Wen Jie Shi, Rong Hui Li, Xu Dong Zhang, Li Ping Wang, Shi Jia Xia, Kai Xi Chen, Jun Jie Zhou, Yang Yang Sun, Jing He, Alisdair R. Fernie, Ying Chun Han, Jun Zhou Li, Yu Heng Shi, Xian Qing Liu, Yuan Yuan Zhang, Jie Luo, Cheng Jin

**Affiliations:** ^1^ School of Breeding and Multiplication (Sanya Institute of Breeding and Multiplication) Hainan University Sanya 572025 China; ^2^ Yazhouwan National Laboratory Sanya 572025 China; ^3^ Hainan Seed Industry Laboratory Sanya 572025 China; ^4^ Sanya Institute Hainan Academy of Agricultural Sciences Sanya 572025 China; ^5^ Max Planck Institute of Molecular Plant Physiology Potsdam‐Golm 14476 Germany; ^6^ Henan Key Laboratory of Rice Molecular Breeding and High Efficiency Production, College of Agronomy Henan Agricultural University Zhengzhou 450046 China

**Keywords:** ABA, cold tolerance, natural variation, stress tolerance, vitamin B6

## Abstract

Vitamin B6 (VB6) is an essential nutrient that must be obtained from the diet by humans and livestock, and plays an important role in response to various biotic and abiotic stresses in plants. Although the VB6 biosynthesis pathway has been uncovered in higher plants, understanding of its genetic control remains limited, and the role of VB6 in responding to cold stress is largely unknown. Here, we showed that variation in VB6 levels in rice is attributable to differential transcription of *OsPDX1.2*, a pyridoxal phosphate synthase, due to polymorphisms in its promoter. Natural variations in the *OsPDX1.2* promoter affect the binding affinity of OsbZIP18, a transcriptional factor repressing *OsPDX1.2* transcription. Loss of function in *OsPDX1.2* or overexpression of *OsbZIP18* significantly reduces cold tolerance in rice. Further investigation revealed that both genes affect cold tolerance by regulating VB6 and abscisic acid (ABA) biosynthesis. Moreover, the frequency of the cold‐tolerant *pOsPDX1.2* ^*Hap2*^ increases at higher latitudes, whereas the cold‐sensitive *pOsPDX1.2* ^*Hap1*^ increases at lower latitudes, indicating that natural variation in *OsPDX1.2* contributes to differential cold adaptation in rice. Overall, our results reveal a regulatory pathway controlling rice VB6 accumulation and cold tolerance via the OsbZIP18–*OsPDX1.2* module, providing a potential target for improving rice quality and stress tolerance.

## Graphical abstract

Allelic variation in the promoter of the pyridoxal phosphate synthase OsPDX1.2 caused variation in the ACE‐box cis‐elements, resulting in differing *OsPDX1.2* expression due to differences in binding affinity between the OsbZIP18 transcription factor and the ACE‐box, contributing to variation in vitamin B6 levels and cold tolerance among rice varieties.

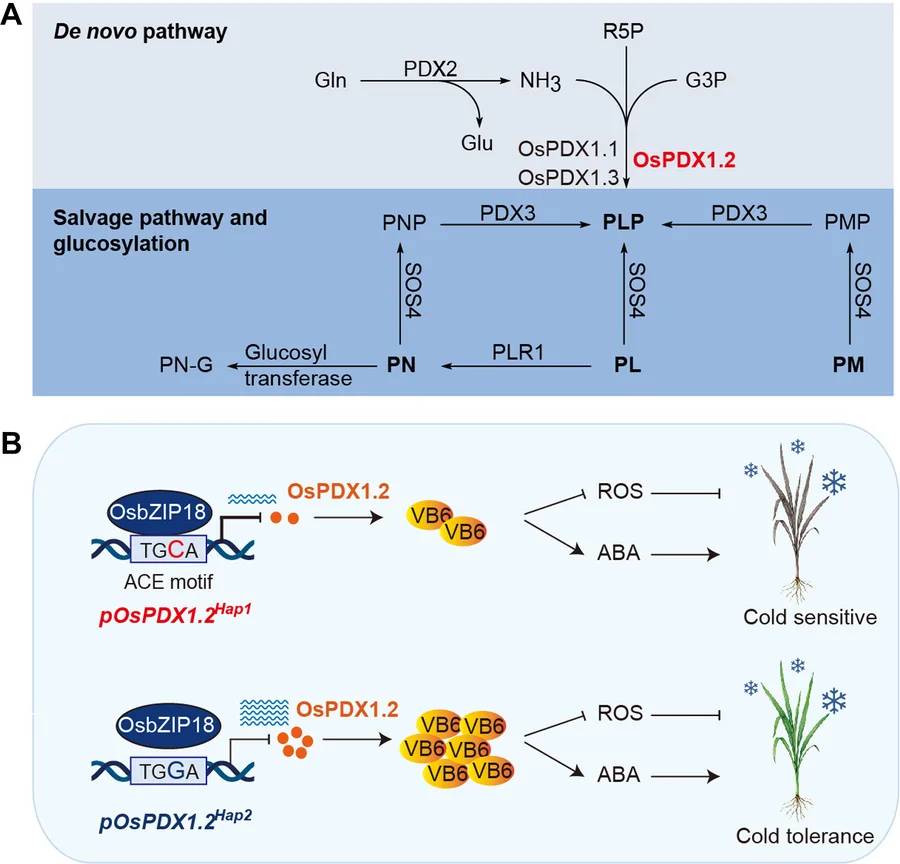

## INTRODUCTION

Vitamin B6 (VB6), a water‐soluble vitamin essential for all living organisms, consists of six related compounds: pyridoxine (PN), pyridoxal (PL), pyridoxamine (PM), and their phosphorylated forms pyridoxine 5′‐phosphate (PNP), pyridoxal 5′‐phosphate (PLP), and pyridoxamine 5′‐phosphate (PMP) (Fitzpatrick et al., 2007; Roje, 2007). PLP acts as a cofactor involved in catalyzing over 140 biochemical reactions, predominantly in amino acids, glucose, and lipid metabolism, surpassing any other known coenzyme in reaction diversity (Percudani and Peracchi, 2003; Mooney and Hellmann, 2010; Colinas et al., 2016). Vitamin B6 cannot be synthesized by humans or animals, and must therefore be acquired from their diet, primarily from plant‐based foods (Fitzpatrick et al., 2012). To date, our understanding of VB6 metabolic regulation in plants remains limited, and yet, it is crucial for biofortification efforts.

In plants, the *de novo* VB6 biosynthesis pathway involves two cytosolic enzymes: PDX1 (pyridoxal phosphate synthase) and PDX2 (Burns et al., 2005). PDX2 catalyzes the release of ammonia from glutamine, which PDX1 subsequently combines with ribose 5‐phosphate and glyceraldehyde 3‐phosphate to synthesize PLP (Tambasco‐Studart et al., 2005, 2007). The rice genome contains three PDX1 homologs (OsPDX1.1, OsPDX1.2, and OsPDX1.3) (Chen et al., 2014; Liu et al., 2022b). Total VB6 levels decreased in *ospdx1.2* knockout plants, but PN and its derivative increased in *OsPDX1.1*‐overexpressing plants, indicating both genes' essential roles in VB6 biosynthesis (Chen et al., 2018; Liu et al., 2022b). However, the biochemical functions of these genes (including *OsPDX1.1*, *OsPDX1.2*, and *OsPDX1.3*) remain uncharacterized in rice. Moreover, to our knowledge, no transcription factors regulating VB6 biosynthesis have been identified in plants.

VB6 functions as a potent reactive oxygen species (ROS)‐scavenging antioxidant and plays a key role in biotic and abiotic stress responses in plants (Danon et al., 2004; Havaux et al., 2009; Leasure et al., 2011; Leuendorf et al., 2014; Moccand et al., 2014; Dell'Aglio et al., 2017; Czegeny et al., 2019; Liu et al., 2022b, 2022c, 2025a). For example, the exogenous application of PN can significantly alleviate salt toxicity symptoms and reduce ROS accumulation in rice (Nan et al., 2020). Recent research showed that the effector protein AvrRxo1 enhances *Xanthomonas oryzae* pv*. oryzicola* (*Xoc*) virulence and suppresses stomatal immunity by targeting and subsequently degrading OsPDX1 (Liu et al., 2022b). In Arabidopsis, *AtPDX1* expression is induced by abiotic stresses such as drought, high light, ozone stress, and UV radiation, whereas the *Atpdx1.3* mutant shows hypersensitivity to salt, high light, photo‐oxidative, and UV‐B stress (Titiz et al., 2006; Wagner et al., 2006; Denslow et al., 2007; Dell'Aglio et al., 2017). Cold stress is a major abiotic stress that adversely affects plant growth and crop productivity. Previous studies indicate that cold stress induces protective proteins and metabolites (e.g., compatible solutes and ROS scavengers) to enhance tolerance (Kaplan et al., 2004; Guy et al., 2007; Zhu et al., 2007; Maruyama et al., 2014; Zhang et al., 2016). Nevertheless, the role of VB6 in cold stress response and its molecular regulatory mechanisms in plants remain largely unknown.

Previous studies demonstrate substantial genetic variation in VB6 accumulation in different tissues among rice cultivars (Chen et al., 2014; Mangel et al., 2022). By using multi‐connected biparental chromosome segment substitution line populations, four quantitative trait loci associated with the accumulation of PN and its derivative have been identified in flag leaves (Chen et al., 2018). However, the natural allelic variation underlying VB6 accumulation remains unknown and requires further investigation.

In this study, we identified OsPDX1.2 as a major determinant of rice VB6 content by combining a metabolite‐based genome‐wide association study (mGWAS) with functional analysis. A natural variant in the *OsPDX1.2* promoter affects its expression by altering binding to the bZIP transcription factor OsbZIP18. Further study revealed that *OsPDX1.2* positively regulates cold tolerance by promoting the synthesis of VB6 and abscisic acid (ABA). Geographical association analysis revealed that the natural variation of *OsPDX1.2* is associated with divergence in cold adaptation among rice varieties.

## RESULTS

### Genetic basis of natural variation in VB6 levels revealed by mGWAS

Using high‐throughput LC‐MS/MS analysis, we identified a total of seven VB6 vitamers and derivatives in the leaves of ZH11 (var. *japonica*, cv Zhonghua 11), namely, pyridoxal (PL), pyridoxamine (PM), pyridoxine (PN), pyridoxal 5′‐phosphate (PLP), pyridoxine *O*‐glucoside (PN‐G), 4‐pyridoxic acid (4PA), and 4‐pyridoxic acid *O*‐glucoside (4PA‐G) (Table S1). The signal intensity of PLP, the biologically active coenzyme form of VB6, was lower than PN and its derivatives (Figure S1). Subsequently, we analyzed the natural variation of PN and its derivatives PN‐G, 4PA, and 4PA‐G in a core collection of 512 rice accessions, comprising 290 *indica*, 151 *japonica*, and 71 other rice cultivars (Figure S2A; Dataset S1). The results revealed substantial variations in the contents of PN, PN‐G, 4PA, and 4PA‐G among the rice cultivars, with coefficients of variation (CV) ranging from 58% to 101% (Figures 1A, S2B–E). A strong positive correlation was observed between PN and PN‐G, 4PA, and 4PA‐G, as well as PN‐G and 4PA‐G (Figure 1B). Among these subpopulations, the *japonica* cultivars demonstrated the highest accumulation of all four vitamers (PN, PN‐G, 4PA, and 4PA‐G), while *indica* cultivars displayed a wide range of variation in the contents of PN and PN‐G (Figures 1C–F, S2F–I).

**Figure 1:**
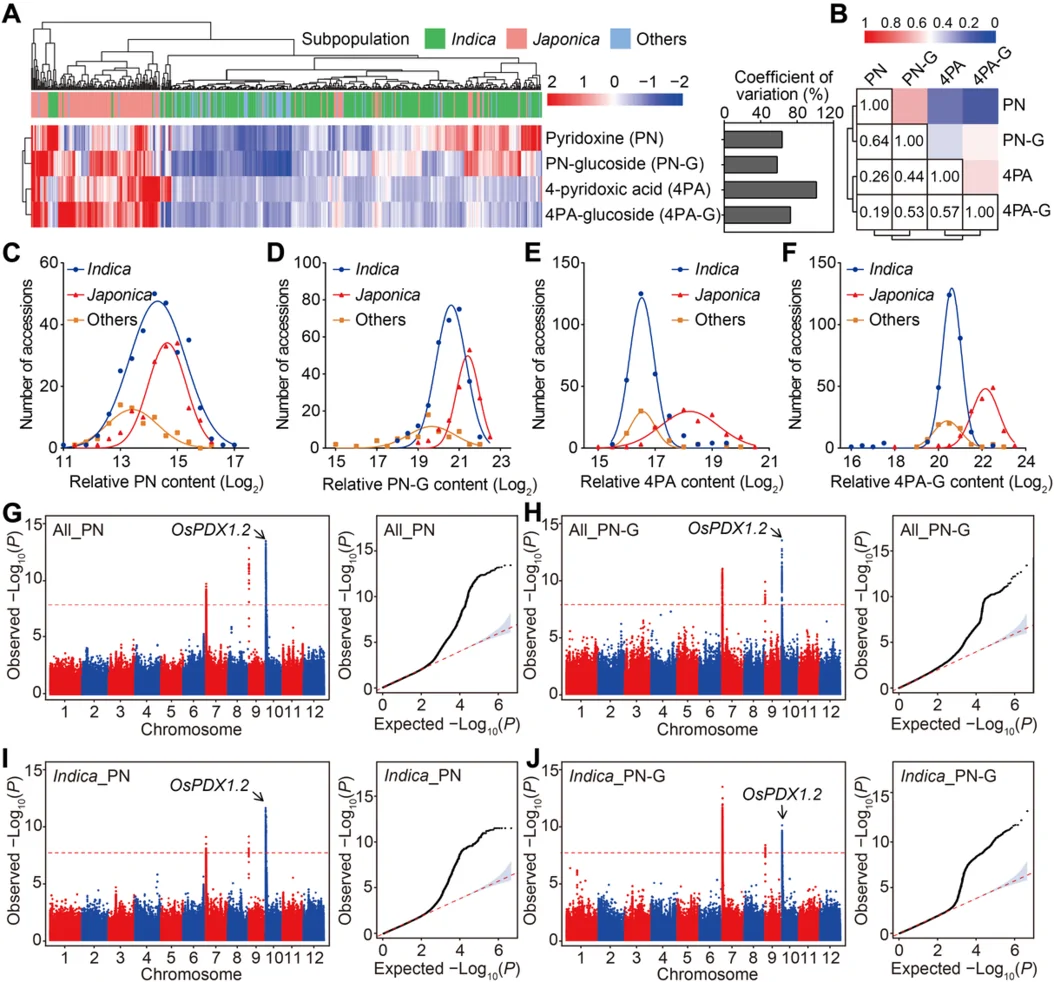
Natural variation of vitamin B6 (VB6) components in rice leaves **(A)** Heatmap of the natural variation and coefficient of variation (CV) of VB6 vitamers in rice. The content value of each VB6 is normalized and hierarchical clustering is performed. The color bar indicates *Z*‐score values, with red and blue representing relatively higher and lower values, respectively. PN, Pyridoxine. PN‐G, pyridoxine *O*‐glucoside. 4PA, 4‐pyridoxic acid. 4PA‐G, 4‐pyridoxic acid *O*‐glucoside. **(B)** Correlations among PN, PN‐G, 4PA, and 4PA‐G. The color bar indicates Pearson s correlation coefficients (*r*). **(C**–**F)** Frequency distribution of the contents of PN **(C)**, PN‐G **(D)**, 4PA **(E)**, and 4PA‐G **(F)** in 512 rice accessions. **(G**–**H)** Manhattan plots of GWAS and quantile–quantile (QQ) plots of PN **(G)** and PN‐G **(H)** in the full population. **(I**, **J)** Manhattan plots of GWAS and QQ plots of PN **(I)** and PN‐G **(J)** in the *indica* subpopulation.

Subsequently, mGWAS were conducted using a mixed linear model (MLM) in the full population, as well as in the *indica* and *japonica* subpopulations. Applying Bonferroni‐corrected genome‐wide significance thresholds (P_LMM_ = 6.6 × 10^−8^ for the full population, 8.7 × 10^−8^ for *indica*, and 2.0 × 10^−7^ for *japonica*), three major loci located on chromosomes 7, 9, and 10 were detected for both PN and PN‐G (Figure 1G–J; Table S2). Notably, these associations were consistently identified in both the full population and the *indica* subpopulation (Figure 1G–J). The phenotypic variation explained by each locus ranged from 6.52% to 10.38%, with the locus located on chromosome 10 demonstrating the strongest effects for both PN and PN‐G in the full population (Table S2).

### Natural variations in *OsPDX1.2* promoters predominantly determine VB6 levels in rice

We further focused our attention on the significant association signal in a 48‐kb genomic interval block (0.044–0.092 Mb) on chromosome 10, which contained five protein‐coding genes, as annotated in the Nipponbare reference genome (Figures 2A, S3A). To ascertain the causal gene underlying this locus, we performed a transcriptome‐wide association study (TWAS) using leaf transcriptome data of 287 rice accessions obtained from a previous study (Liu et al., 2022a). This analysis refined the candidate set from the initial five genes to a single candidate, LOC_Os10g01080 (*OsPDX1.2*), a pyridoxine synthase belonging to the SOR/SNZ family, whose expression level showed a positive correlation with both PN and PN‐G accumulation (Figures 2B–E, S3B–E).

**Figure 2:**
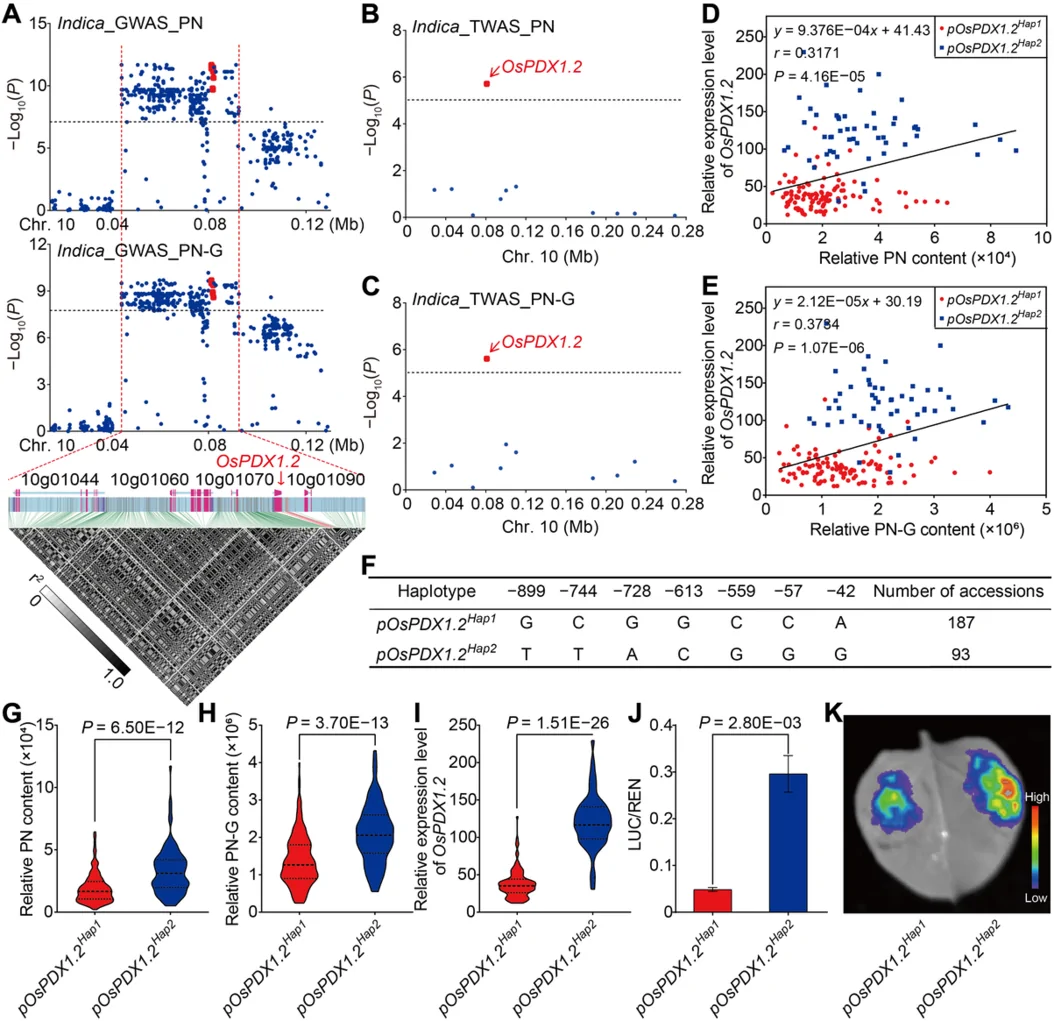
**Natural variations in** ***OsPDX1*** **.2 are associated with VB6 accumulation in rice** **(A)** Local Manhattan plots of GWAS results of PN and PN‐G in the *indica* subpopulation. The corresponding linkage disequilibrium (LD) heatmap for the candidate region (demarcated by red dashed lines) is shown below. The candidate interval encompassing the peak is indicated by red vertical lines, while red dots represent SNPs within the promoter region of *OsPDX1.2*; these corresponding SNPs are also highlighted with red lines in the LD heatmap. **(B**, **C)** Local transcriptome‐wide association study (TWAS) for PN **(B)** and PN‐G **(C)**. Red dots indicate association signals corresponding to *OsPDX1.2*. **(D**, **E)** Correlation between *OsPDX1.2* expression and levels of PN **(D)** and PN‐G **(E)** in 161 *indica* accessions. The *r* values were derived from Pearson correlation analysis. **(F)** Haplotype analysis of the polymorphic promoter region of *OsPDX1.2* in the *indica* subpopulation. **(G**–**I)** Comparative analysis of PN **(G)**, PN‐G **(H)**, and *OsPDX1.2* expression level **(I)** between two distinct haplotypes. *P* values were calculated using the Wilcoxon rank‐sum test. **(J**, **K)** Comparison of promoter activity between different haplotypes of *OsPDX1.2*. The relative luciferase activities measured by the dual‐luciferase reporter assay system. Data are shown as the means ± *SE* (*n* = 3). Statistical significance was determined by two‐sided Student's *t*‐tests.

To investigate the contribution of natural variation in *OsPDX1.2* to the variations of PN and PN‐G levels in rice leaves, we conducted a haplotype analysis of *OsPDX1.2* in both the full population and the *indica* subpopulation. Seven single‐nucleotide polymorphisms (SNPs) were identified in the promoter region of *OsPDX1.2*, whereas no nucleotide substitutions were detected within its coding sequence (CDS) (Figure S3A; Table S3). We therefore classified the full population into four haplotypes according to the variation in the *OsPDX1.2* promoter region (Figure S3F). Notably, the *indica* subspecies contains two major haplotypes (*pOsPDX1.2* ^*Hap1*^ and *pOsPDX1.2* ^*Hap2*^), whereas *pOsPDX1.2* ^*Hap1*^ is absent in *japonica* rice (Figures 2F, S3F). The rice accessions carrying the *pOsPDX1.2* ^*Hap1*^ allele showed significantly lower levels of PN, PN‐G, and *OsPDX1.2* expression than *pOsPDX1.2* ^*Hap2*^ (Figures 2G–I, S3G–I). Transient expression assays in *Nicotiana benthamiana* leaves revealed that the promoter segments from *pOsPDX1.2* ^*Hap2*^ showed 6.2‐fold higher activity than those from *pOsPDX1.2* ^*Hap1*^ (Figure 2J, K). These results provide support for the impact of natural *OsPDX1.2* promoter variations on the transcription of this gene.

### OsPDX1.2 positively regulates the levels of VB6

The expression pattern of *OsPDX1.2* at different growth stages was next investigated with real‐time quantitative PCR (RT‐qPCR), and the results showed that *OsPDX1.2* is predominantly expressed in leaves during the vegetative growth stage (Figure 3A). Subcellular localization assays using transient expression of 35S:eGFP–OsPDX1.2 in *N. benthamiana* leaves revealed that the fusion protein localizes predominantly to the cytoplasm (Figure 3B), which was further confirmed by similar results obtained in rice protoplasts (Figure S4). *In vitro* assays demonstrated that the OsPDX1.2 enzyme catalyzes the direct synthesis of PLP from ribose 5‐phosphate (R5P), glyceraldehyde 3‐phosphate (G3P), and glutamine (Gln), thereby confirming the role of OsPDX1.2 as a functional PLP synthase (Figure 3C, D). To further investigate the function of OsPDX1.2 in VB6 metabolism, we generated *ospdx1.2* knockout mutants (*ospdx1.2‐1*, *ospdx1.2‐2*, and *ospdx1.2‐3*) using the CRISPR/Cas9 system (Figure S5A). Metabolite profiling revealed a significant reduction in the accumulation of VB6 vitamers in the leaves and seeds of *ospdx1.2* mutants compared to the wild type (Figures 3E–I, S5B–F). Conversely, *OsPDX1.2*‐overexpressing plants showed significantly increased accumulation of VB6 vitamers in leaves compared to the wild type (Figure S6). These results collectively indicate that OsPDX1.2 encodes a functional PLP synthase and positively regulates VB6 biosynthesis in rice.

**Figure 3:**
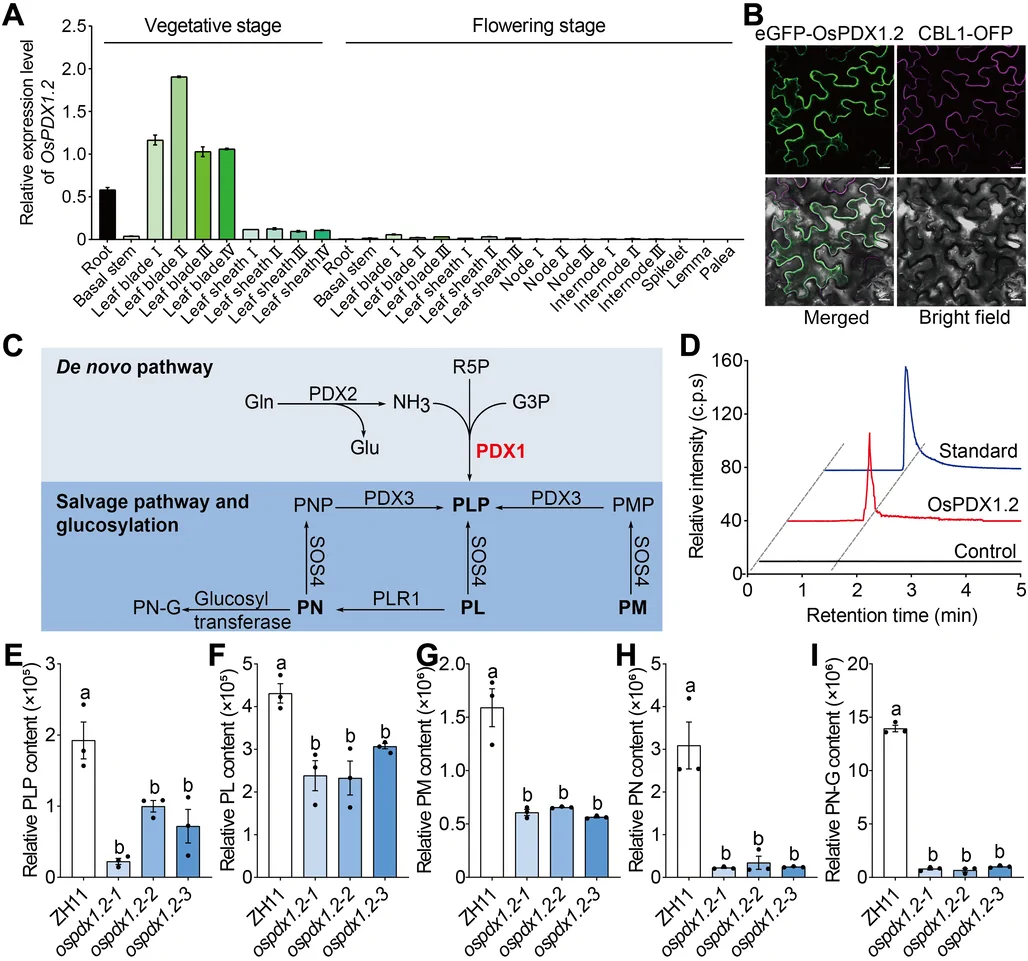
**Functional characterization of** ***OsPDX1.2*** **in rice** **(A)** Relative expression levels of *OsPDX1.2* in different tissues at various growth stages. **(B)** Subcellular localization of OsPDX1.2. CBL1‐OFP was used as a plasma membrane marker. Scale bar = 20 μm. **(C)** Schematic of VB6 biosynthesis and metabolism in plants. **(D)** *In vitro* enzyme activity assays of OsPDX1.2 protein. **(E**–**I)** Relative content of PLP **(E)**, PL **(F)**, PM **(G)**, PN **(H)**, and PN‐G **(I)** in leaves of wild‐type and *ospdx1.2* mutant plants. Data are shown as the means ± *SE* (*n* = 3). Statistical significance was determined by one‐way ANOVA (Tukey's test).

### OsPDX1.2 positively regulates cold tolerance in rice seedlings

Previous studies have shown that the expression of *PDX1* genes is induced by various abiotic stresses, including high light, drought, and UV radiation in plants (Denslow et al., 2007; Titiz et al., 2006). The expression of *OsPDX1.2* was significantly upregulated by cold stress (Figure S7), suggesting that it may also play an important role in the rice cold stress response. To test this hypothesis, we analyzed the phenotype of the *ospdx1.2* mutant under cold stress. Under normal growth conditions, no obvious phenotypic differences were observed between *ospdx1.2* mutants and the wild type (Figure 4A). However, after 5 d of cold stress treatment (4°C), both *ospdx1.2* mutants were more susceptible to cold stress, displaying significantly lower biomass and survival rates than the wild type after a 7‐d recovery at 25°C (Figure 4C–E). Consistent with this observation, histochemical staining using 3,3‐diaminobenzidine (DAB) and nitrotetrazolium blue chloride (NBT) showed enhanced reactive oxygen species (ROS) accumulation in the leaves of *ospdx1.2* mutants after cold stress (Figure 4F). The levels of malondialdehyde (MDA) in *ospdx1.2* mutants were significantly increased under cold stress (Figure 4B). Conversely, *OsPDX1.2*‐overexpressing plants showed enhanced cold tolerance, with higher biomass, survival rate, and reduced ROS accumulation and MDA levels compared to the wild type under cold stress (Figure S8). Furthermore, exogenous application of PN significantly rescued the cold sensitivity of rice seedlings (Figure 4), suggesting that *OsPDX1.2* positively regulates cold tolerance by regulating VB6 synthesis at the seedling stage.

**Figure 4:**
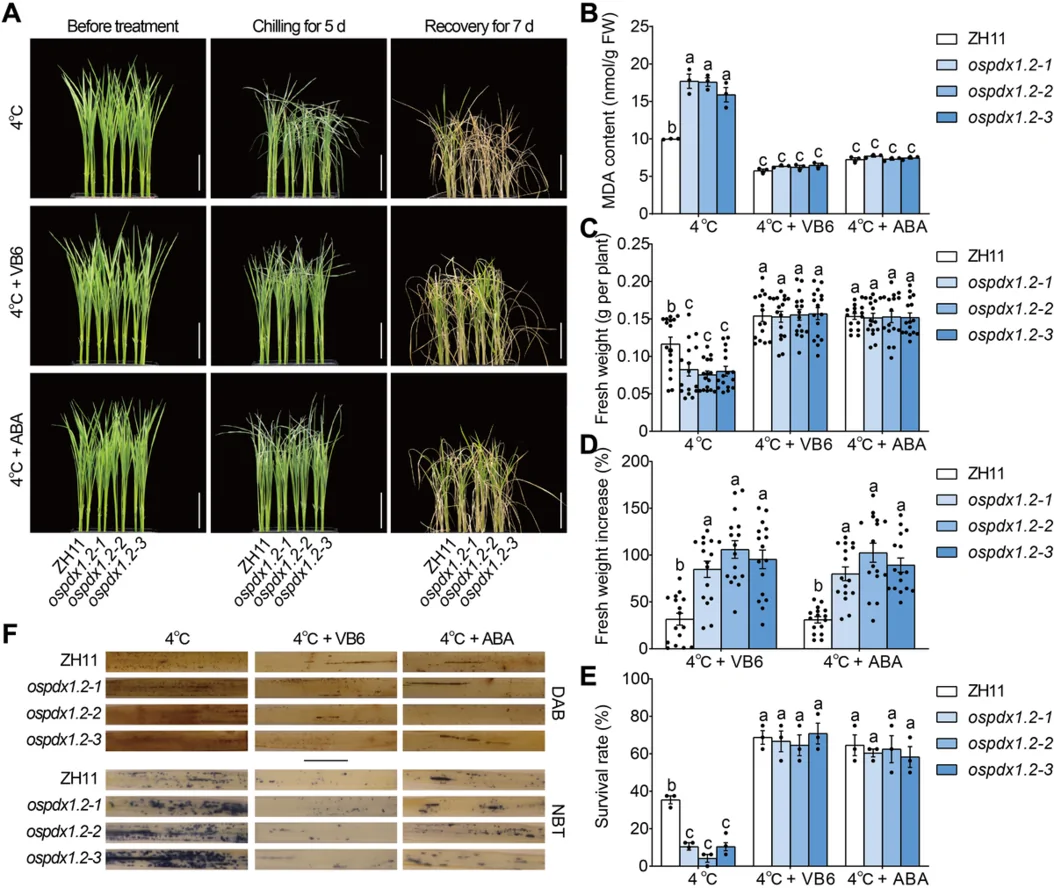
**Exogenous VB6 and ABA application rescues the cold‐sensitive phenotype of the** ***ospdx1.2*** **mutant** **(A)** Phenotypic analysis of cold tolerance in wild‐type and *ospdx1.2* mutant plants with exogenous VB6 and ABA application. Twelve‐d‐old plants grown under normal conditions were sprayed daily with 30 μM PN (VB6), 30 μM ABA, or distilled water (control) during the 5‐d treatment at 4°C, followed by a 7‐d recovery at 25°C. Scale bar, 5 cm. **(B)** MDA contents in leaves of 12‐d‐old *ospdx1.2* transgenic and wild‐type rice seedlings after a 24‐h treatment at 4°C. **(C**–**E)** Fresh weight **(C)**, fresh weight increase **(D)**, and survival rate **(E)** of plants measured after recovery from the 4°C treatment. **(F)** Histochemical analysis of reactive oxygen species (ROS) in leaves under cold stress. Hydrogen peroxide (H_2_O_2_) and superoxide anion (O_2_^−^) accumulations were detected using diaminobenzidine (DAB) and nitroblue tetrazolium (NBT) staining, respectively. Seedlings were treated at 4°C for 24 h prior to staining. Scale bar, 1 cm. In **(B**, **E)**, data are presented as means ± *SE* (*n* = 3). In **(C**, **D)**, data are presented as means ± *SE* (*n* = 16). Statistical significance for panels **(B**–**E)** was determined by one‐way ANOVA (Tukey's test).

Recent studies indicate that VB6 is required as a cofactor in the biosynthesis of abscisic acid (ABA), which positively regulates the cold stress response in plants (Ji et al., 2011; Liu et al., 2022b; Li et al., 2025b). This occurs because VB6 acts as an ABA3 (molybdenum cofactor sulfurase) cofactor that converts molybdenum cofactor into its sulfurated form, which is in turn essential for aldehyde oxidase AAO3 to catalyze the conversion of abscisic aldehyde into ABA (Seo et al., 2000, 2001; Bittner et al., 2001). A significant decrease in ABA accumulation was detected in the leaves of *ospdx1.2* mutants (Figure S9A), suggesting that the cold stress‐sensitive phenotype was likely attributable to an ABA deficiency. In contrast, the ABA content was significantly increased in *OsPDX1.2*‐overexpressing plants compared to the wild type (Figure S9B). Exogenous ABA complemented the cold‐sensitive phenotype of the *ospdx1.2* mutants (Figure 4). These results indicate that *OsPDX1.2* potentially confers cold tolerance probably via the regulation of both VB6 and ABA biosynthesis.

### Natural variation in *OsPDX1.2* contributes to adaptation to cold stress in rice

Given that OsPDX1.2 can regulate cold tolerance in the ZH11 cultivar, we explored whether its natural variation is associated with cold stress response in rice populations. A subset of 36 randomly selected accessions (18 *pOsPDX1.2* ^*Hap1*^ and 18 *pOsPDX1.2* ^*Hap2*^) from the *indica* subpopulation was evaluated for cold tolerance to minimize confounding effects from genetic background variation across subpopulations. The results showed that *pOsPDX1.2* ^*Hap2*^ plants had enhanced cold tolerance compared to *pOsPDX1.2* ^*Hap1*^ plants, accompanied by increased PN and PN‐G contents, elevated *OsPDX1.2* expression, and improved survival rates under cold stress (Figures 5A–D, S10). Furthermore, we found that OsPDX1.2 showed a strong association with latitude, with *pOsPDX1.2* ^*Hap2*^ occurring in higher latitudes, while *pOsPDX1.2* ^*Hap1*^ was more frequently distributed in lower latitudes (Figure 5E–G; Dataset S2). These data suggest that *pOsPDX1.2* ^*Hap2*^ may facilitate adaptation to cold environments in rice.

**Figure 5:**
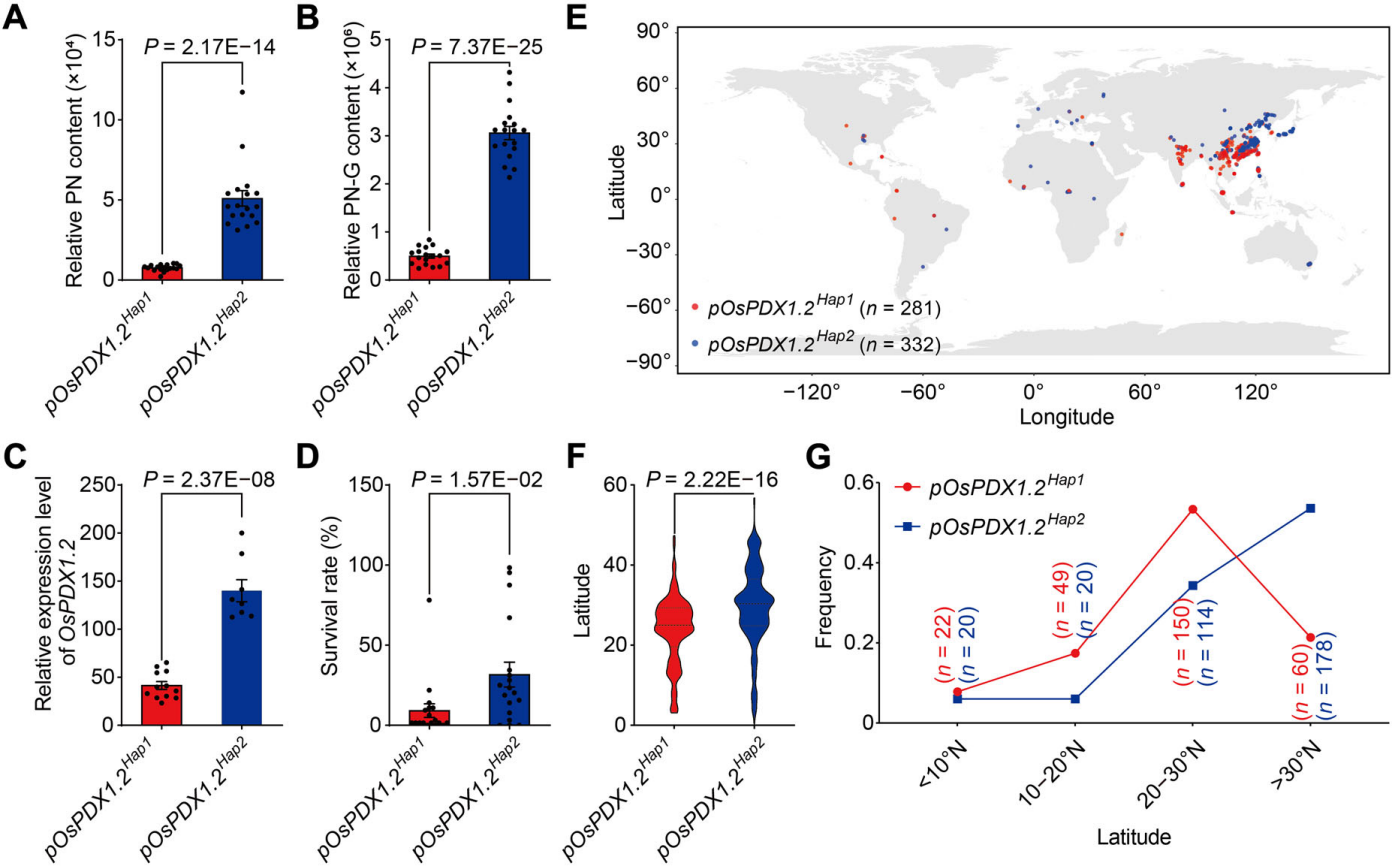
**Natural variation of** ***OsPDX1.2*** **is associated with cold tolerance and latitudinal adaptation in rice** **(A**–**C)** Comparative analysis of PN **(A)**, PN‐G **(B)**, and *OsPDX1.2* transcript levels **(C)** between the *pOsPDX1.2* ^*Hap1*^ and *pOsPDX1.2* ^*Hap2*^ haplotypes in 36 *indica* accessions. **(D)** Survival rates of *pOsPDX1.2* ^*Hap1*^ and *pOsPDX1.2* ^*Hap2*^ under cold stress in the *indica* rice population. Eight‐d‐old seedlings were exposed to 4°C for 4 d and then recovered at 25°C for 7 d. **(E)** Global distribution of 709 cultivated rice accessions carrying the *pOsPDX1.2* ^*Hap1*^ and *pOsPDX1.2* ^*Hap2*^ alleles. **(F)** Latitude distribution of *pOsPDX1.2* haplotypes in 709 rice accessions. *P* values were calculated using the Wilcoxon rank‐sum test. **(G)** The line chart depicts the relationship between allele frequency and latitude for the *pOsPDX1.2* haplotypes. In **(A**–**D)**, data are presented as means ± *SE* (*n* = 18). Statistical significance was determined by two‐sided Student's *t*‐tests.

### OsbZIP18 negatively regulates cold tolerance in rice

Since natural variation in the *OsPDX1.2* promoter contributes to the difference in *OsPDX1.2* gene expression and VB6 accumulation among rice varieties (Figure 2D–K), we compared the *cis*‐acting elements in *pOsPDX1.2* ^*Hap1*^ and *pOsPDX1.2* ^*Hap2*^ promoter regions. The results showed that a guanine (G) to cytosine (C) point mutation at the −559 position leads to the gain of an ACE *cis*‐element (ACGT) (Figure 6A), a well‐characterized canonical binding site for bZIP transcription factors (Gangappa and Botto, 2016; Dröge‐Laser and Weiste, 2018; Dröge‐Laser et al., 2018). Therefore, we performed a co‐expression analysis using the public database ATTED‐II (https://atted.jp/top_search/#CoExSearch), which identified *OsbZIP18* as a candidate (Table S4).

**Figure 6:**
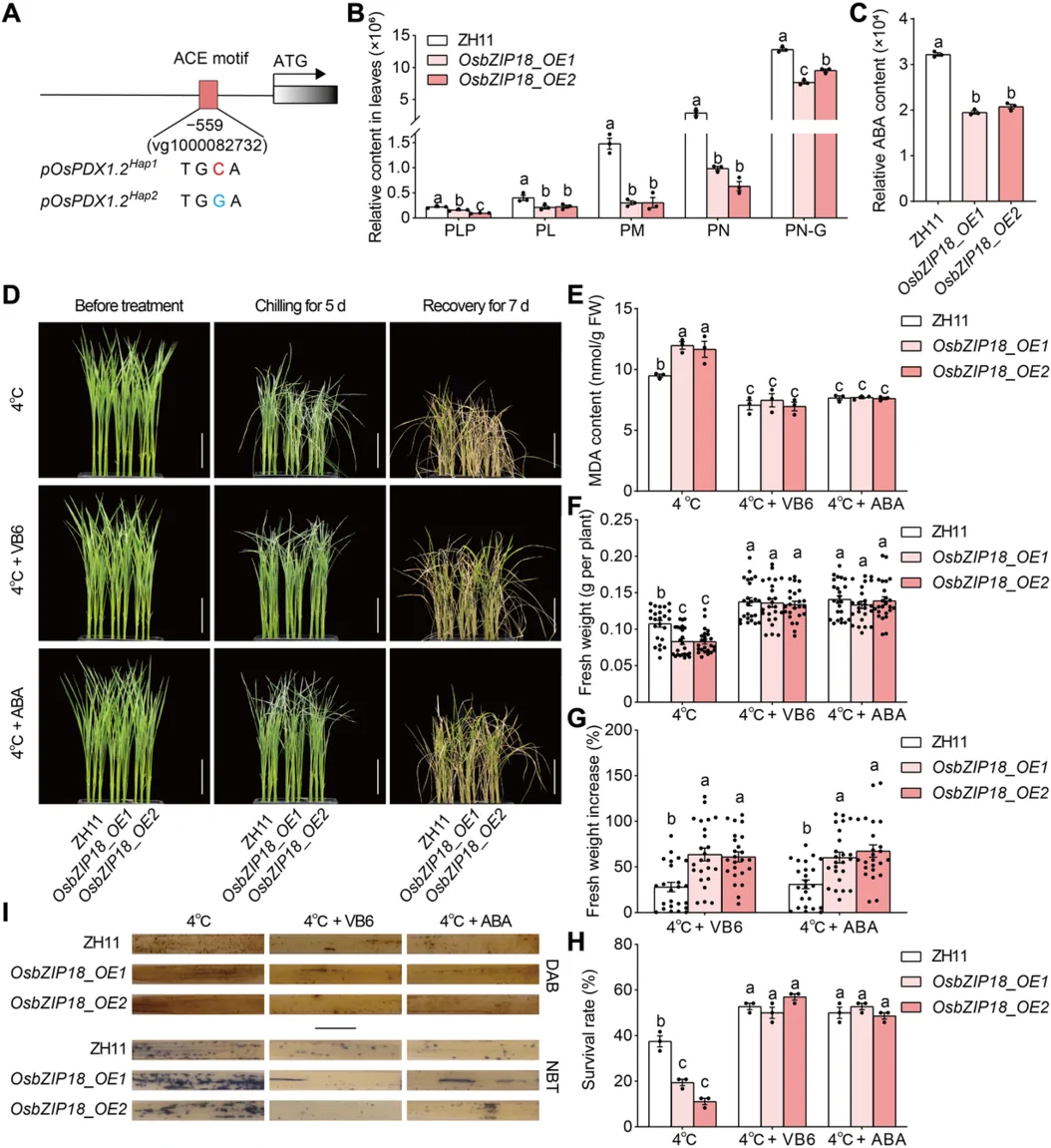
**Exogenous applications of VB6 and ABA enhance cold stress tolerance in** ***OsbZIP18*** **‐overexpressing rice plants** **(A)** Diagram of the *pOsPDX1.2* ^*Hap1*^ and *pOsPDX1.2* ^*Hap2*^ regions. Pink rectangles indicate ACE *cis*‐elements, known binding sites for bZIP transcription factors. The SNP (vg1000082732) that distinguishes the two haplotypes is highlighted in red. **(B**, **C)** Vitamin B6 **(B)** and ABA **(C)** contents in leaves of wild‐type and *OsbZIP18‐*overexpressing (OE) plants. **(D)** Phenotypic analysis of cold tolerance in wild‐type and *OsbZIP18*_*OE* plants following exogenous VB6 and ABA supplementation. Twelve‐d‐old seedlings grown under standard conditions received daily foliar sprays of 30 μM PN (VB6), 30 μM ABA, or distilled water (control) throughout the 5‐d cold treatment at 4°C, after which plants were transferred to 25°C for a 7‐d recovery period. Scale bar, 5 cm. **(E)** MDA contents in leaves of 12‐d‐old *OsbZIP18_OE* transgenic and wild‐type seedlings after a 24‐h treatment at 4°C. **(F**–**H)** Fresh weight **(F)**, fresh weight increase **(G)**, and survival rate **(H)** of plants measured after recovery from cold stress. **(I)** Accumulation of reactive oxygen species in cold‐stressed leaves, visualized by DAB (for H_2_O_2_) and NBT (for O_2_^−^) staining. Scale bar, 1 cm. In **(E**, **H)**, data are presented as means ± *SE* (*n* = 3). In **(F**, **G)**, data are presented as means ± *SE* (*n* = 24). Statistical significance was determined by one‐way ANOVA (Tukey's test).

To determine whether OsbZIP18 is involved in regulating VB6 accumulation and the cold stress response, we generated *OsbZIP18*‐overexpressing transgenic rice lines. Consistently, *OsbZIP18_OE* plants displayed markedly reduced levels of VB6 vitamers, ABA as well as diminished cold tolerance compared to the wild type (Figure 6B, C). Conversely, no significant differences in *OsPDX1.2* expression, VB6 levels, or ABA content were detected between the *osbzip18* mutant and the wild type (Figure S11), which may be explained by functional redundancy between OsbZIP18 and OsbZIP48 (Burman et al., 2018; Xiong et al., 2024). Moreover, the application of VB6 and ABA dramatically significantly alleviated the cold sensitivity of the *OsbZIP18_OE* plants mutants (Figure 6D–I). These results demonstrate that OsbZIP18 regulates cold tolerance by modulating the biosynthesis of VB6 and ABA.

### Natural variations in the *OsPDX1.2* promoter affect the binding of OsbZIP18

To investigate whether *OsPDX1.2* is a target of OsbZIP18, we first analyzed the expression level of *OsPDX1.2* in *OsbZIP18_OE* plants. Indeed, *OsPDX1.2* expression was significantly downregulated in *OsbZIP18_OE* plants (Figure 7A). Yeast one‐hybrid (Y1H) assays and electrophoretic mobility shift assays (EMSAs) confirmed the ability of OsbZIP18 to bind the ACE motif (Figure 7B, C). These results indicate that OsbZIP18 acts as a negative regulator of *OsPDX1.2*.

**Figure 7:**
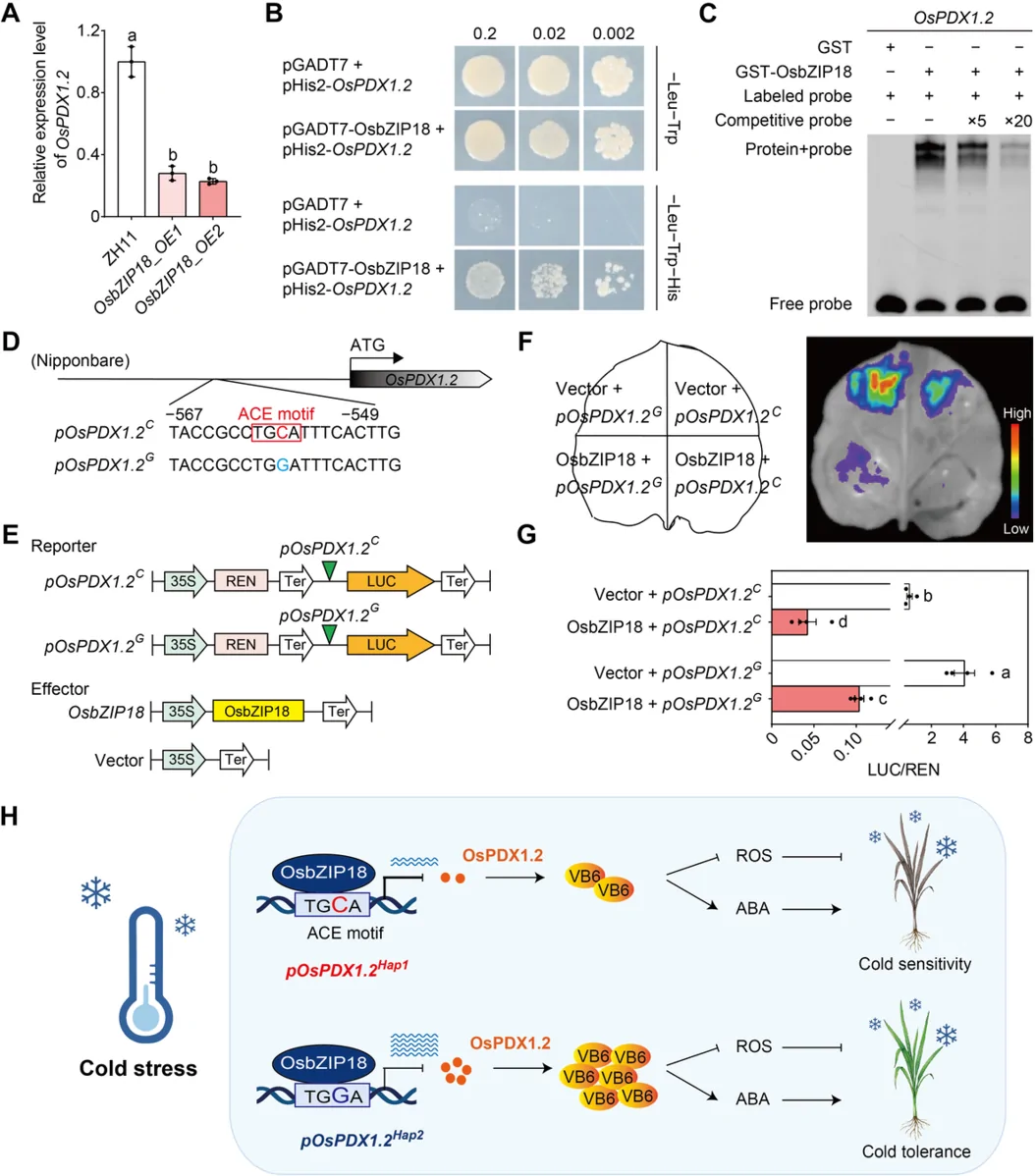
The OsbZIP18–*OsPDX1.2* regulatory module underlies natural variation in rice cold tolerance **(A)** Transcript levels of *OsPDX1.2* in leaves of *OsbZIP18_OE* plants. The relative expression of *OsPDX1.2* was measured by RT–qPCR normalized to *Ubiquitin*. Data are shown as the means ± *SE* (*n* = 3). Statistical significance was determined by one‐way ANOVA (Tukey's test). **(B)** Yeast one‐hybrid assay demonstrating the binding of OsbZIP18 to the *OsPDX1.2* promoter. The combination of empty pGADT7 and pHIS2‐*OsPDX1.2* was co‐transformed as a negative control. **(C)** Electrophoretic mobility shift assay (EMSA) of OsbZIP18 binding to the ACE motif in the *OsPDX1.2* promoter. Competitive binding assays were performed using five‐ and 20‐fold molar excess of unlabeled probe. **(D)** Diagram of the site‐directed mutagenesis locations in the *OsPDX1.2* promoter region. **(E)** Schematic representation of the effector and reporter constructs for transient expression in *N. benthamiana* leaf epidermis. **(F**, **G)** Transcriptional repression of *pOsPDX1.2* ^*C*^ and *pOsPDX1.2* ^*G*^ by OsbZIP18. Data are shown as the means ± *SE* (*n* = 4). Statistical significance was determined by two‐sided Student's *t*‐tests. **(H)** A proposed model for natural polymorphism in OsPDX1.2 regulating cold tolerance through vitamin B6. The transcription factor OsbZIP18 represses *OsPDX1.2* expression and vitamin B6 accumulation. Natural variation in the promoter ACE element modulates this regulation: Stronger OsbZIP18 binding to the *pOsPDX1.2* ^*Hap1*^ allele more strongly suppresses *OsPDX1.2*, thereby altering ROS and ABA homeostasis and resulting in enhanced cold sensitivity in *pOsPDX1.2* ^*Hap1*^ lines compared to *pOsPDX1.2* ^*Hap2*^ lines. Arrows and T‐bars denote promotion and inhibition, respectively.

Subsequently, dual‐luciferase assays were conducted to determine the transactivation ability of OsbZIP18 with respect to the *OsPDX1.2* promoters. A G‐to‐C point mutation was introduced at the −559 bp position of the *OsPDX1.2* promoter in the background of Nipponbare (*pOsPDX1.2* ^*Hap2*^) via site‐directed mutagenesis, generating the *pOsPDX1.2* ^*C*^ allele, while the original allele was designated as *pOsPDX1.2* ^*G*^ (Figure 7D). After co‐transforming 35S:OsbZIP18 with the *pOsPDX1.2* ^*G*^ or *pOsPDX1.2* ^*C*^ constructs in *N. benthamiana* leaves, the activity of both promoters was suppressed, as compared with that of the vector control (Figure 7E–G). Moreover, the luciferase activity driven by the *pOsPDX1.2* ^*C*^ promoter was substantially lower than that of the *pOsPDX1.2* ^*G*^ promoter (Figure 7F, G), indicating that OsbZIP18 represses the activity of the *OsPDX1.2* promoter, especially that of *pOsPDX1.2* ^*C*^.

## DISCUSSION

Improving quality and stress tolerance is a key goal in crop breeding particularly now, given the dramatic challenges imposed by climate change (Tao et al., 2025). VB6 is an essential nutrient for humans and plays a crucial role in mediating tolerance to both biotic and abiotic stresses in plants (Fitzpatrick et al., 2012; Fudge et al., 2017; Liu et al., 2022d; Fitzpatrick, 2024). Understanding the molecular mechanisms underlying VB6 accumulation and its genetic bases is crucial for developing both VB6‐rich and stress‐tolerant crop varieties. In this study, we identified OsPDX1.2 as a key regulator of VB6 accumulation and cold tolerance in rice. We discovered that sequence variation at SNP‐559 in the *OsPDX1.2* promoter determines the binding affinity of OsbZIP18 and is responsible for the diverse *OsPDX1.2* expression levels and cold tolerance variation in rice population. This study uncovers a novel regulatory module governing VB6 accumulation and cold tolerance in rice (Figure 7H).

We provide a series of evidence demonstrating that OsPDX1.2 in rice (i) is essential for VB6 synthesis and cold tolerance; (ii) regulates cold tolerance by mediating VB6 and ABA accumulation; and (iii) is transcriptionally regulated by OsbZIP18, which is critical for cold responses. *Cis*‐regulatory polymorphisms in the *OsPDX1.2* promoter fine‐tune its expression and correlate with natural variations in VB6 levels and cold tolerance. Collectively, these findings establish OsPDX1.2 as a key regulator of VB6 synthesis, elucidate the regulatory mechanism underlying VB6 metabolism and stress tolerance, and provide a basis for novel rice breeding strategies to develop vitamin B6‐enriched, stress‐tolerant crops supporting sustainable food security.

We determined that the cold tolerance regulated by *OsPDX1.2* depends on the accumulation of VB6 and ABA. Several secondary metabolites, such as proline, branched‐chain amino acids (BCAA), vitamin B5, and VB6, act as effective reactive oxygen species (ROS) scavengers, providing protection against damage induced by stresses, including salt and drought (Mooney and Hellmann, 2010; Kaur and Asthir, 2015; Batista‐Silva et al., 2019; Foyer et al., 2020; Sun et al., 2024). Recent research showed that amino acid metabolism contributes to cold tolerance through a ZmICE1‐dependent mechanism in maize (Jiang et al., 2022). Here, we demonstrate that VB6 enhances rice cold tolerance by scavenging ROS. ABA, an important plant hormone regulating responses to various abiotic stresses, including low temperature, has been shown in numerous studies to significantly enhance cold tolerance in diverse plant species (Baron et al., 2012; An et al., 2022; Parwez et al., 2022; Guan et al., 2023; Zheng et al., 2023; Liu et al., 2024a). VB6 is essential for aldehyde oxidase activity, which catalyzes the final step of ABA biosynthesis (Seo et al., 2000; Bittner et al., 2001; Liu et al., 2022b). Exogenous application of VB6 and ABA rescued the cold stress‐sensitive phenotype of the *ospdx1.2* mutant plants (Figure 4). This finding, together with the observed reductions in VB6 and ABA content in *ospdx1.2* mutants under cold stress, collectively suggests that OsPDX1.2 regulates cold tolerance through VB6 and ABA synthesis. Additionally, the *ospdx1.2* mutant shows suppressed ABA synthesis and altered expression of signaling pathway genes (Figure S12), suggesting that both ABA synthesis and ABA signaling may be involved in *OsPDX1.2*‐mediated cold tolerance. Nevertheless, further verification is required to establish whether both VB6 and ABA are essential for *OsPDX1.2*‐mediated cold tolerance.

To our knowledge, no transcription factors that regulate VB6 biosynthesis have been identified in plants. OsbZIP18 is a homolog of AtHY5, a transcription factor known to promote photomorphogenesis and positively regulate cold adaptation and ultraviolet‐B (UV‐B) radiation tolerance in Arabidopsis (Gangappa and Botto, 2016). In tomato, HY5 positively regulates cold tolerance through the C‐repeat binding factor (CBF) signaling pathway (Zhang et al., 2020). Recent research demonstrated that maize HY5 positively regulates cold tolerance in maize by negatively regulating *COOL1* expression (Zeng et al., 2025). Our previous results demonstrate that OsbZIP18 positively regulates BCAA and serotonin biosynthesis, while negatively regulating rice tolerance to UV‐B (Sun et al., 2020, 2022; Liu et al., 2024b). We revealed here that OsbZIP18 directly inhibits *OsPDX1.2* expression, which in turn suppresses VB6 biosynthesis and reduces cold tolerance. However, knockout of *OsbZIP18* did not affect VB6 content or ABA levels in rice. Given the high amino acid sequence similarity between OsbZIP18 and OsbZIP48 (Burman et al., 2018; Xiong et al., 2024), we speculate that these two genes are functionally redundant in regulating cold tolerance. OsbZIP18 and its functional counterpart HY5 in Arabidopsis, tomato, and maize play opposing roles in response to cold and UV‐B stress, indicating evolutionary differentiation in gene function. Further research is, however, required to determine whether HY5 also regulates the synthesis of VB6 in other plant species. A number of genes have been identified that impart cold tolerance in diverse rice subspecies (Saito et al., 2010; Zhang et al., 2017; Li et al., 2021; Lou et al., 2022). Recent research showed that natural variants of OsbZIP73, OsCTF1, OsCTB3, OsCTB5, and OsTCP19 facilitate cold climate adaptation in *japonica* rice (Liu et al., 2018; Dong et al., 2025; Guo et al., 2025; Li et al., 2025a). Natural variation analyses revealed that *pOsPDX1.2* ^*Hap2*^ conferred higher cold tolerance and enhanced VB6 accumulation compared to *pOsPDX1.2* ^*Hap1*^. These results define *pOsPDX1.2* ^*Hap2*^ as an elite haplotype that enhances VB6 accumulation and confers cold adaptation in rice. Moreover, we previously identified an elite haplotype of *OsbZIP18* associated with reduced transcriptional levels (Sun et al., 2020). The elite variants in OsPDX1.2 and OsbZIP18 may serve as prime targets for gene editing or marker‐assisted breeding to develop rice with both high VB6 content  and stress tolerance.

## MATERIALS AND METHODS

### Plant materials

The 512 rice accessions used in this investigation are detailed in Dataset S1, including variety names, country of origin, geographical coordinates (longitude and latitude), and subpopulation classifications. The *ospdx1.2* mutant was obtained via CRISPR‐Cas9 technology, and the *OsPDX1.2*‐overexpression line was generated by introducing the *OsPDX1.2* coding sequence into the PJC034 vector, followed by *Agrobacterium*‐mediated transformation (Miao et al., 2013). Both the *osbzip18* mutant and the *OsbZIP18*‐overexpression line were obtained from our previous work (Sun et al., 2022).

### Metabolic profiling analysis

Metabolites were extracted and detected as previously described (Shi et al., 2023). Briefly, leaf tissue from 4‐week‐old rice plants and mature seeds was freeze‐dried and then ground into a powder using a mixer mill (MM400, Retsch) at 30 Hz for 1.5 min. 100 mg of the powder was weighed and extracted at 4°C with 70% aqueous methanol containing 0.1 mg/mL lidocaine as an internal standard. After centrifugation and filtration, the extracts were analyzed using LC‐MS/MS (AB SCIEX QTRAP6500). For each transgenic material, at least three independent transgenic lines were selected for targeted metabolite analysis.

### Genome‐wide association studies and transcriptome‐wide association studies

Genome‐wide association analysis was performed as previously described (Chen et al., 2014). In brief, genome‐wide SNPs were obtained from the RiceVarMap database. SNPs with a minor allele frequency (MAF) ≥ 0.05 and present in at least six accessions were retained for association analysis. A linear mixed model implemented in the FaST‐LMM program was used to control for population structure by incorporating a kinship matrix as a random effect. The genome‐wide significance thresholds were set at a nominal level of 0.05, corresponding to *P* = 1.16 × 10^−8^ for the entire population and to *P* = 1.78 × 10^−8^ for the *indica* subpopulation.

Transcriptome‐wide association analysis was performed as described (Li et al., 2024). Specifically, normalized expression values were transformed to a 0–2 scale. Associations between these transformed expression values and phenotypic measurements were tested using the compressed mixed linear model (CMLM) in the GAPIT software package (version 3.1). A Bonferroni correction for the total number of tested gene expression features was applied to determine statistical significance at α = 0.05.

### RNA extraction and expression analyses

Total RNA from rice tissues was extracted using TRIzol reagent (Invitrogen) according to the manufacturer's instructions. First‐strand cDNA synthesis was performed with 3 μg of RNA using the EasyScript One‐Step gDNA Removal and cDNA Synthesis SuperMix Kit (TransGen), following the standard protocol. Quantitative real‐time PCR analyses were conducted on an ABI 7500 Real‐Time PCR system (Applied Biosystems) with the SYBR Premix Ex Taq kit (TOLOBIO). Gene expression levels were normalized using the rice *UBIQUITIN* gene as an internal control. The primers used for these constructs are detailed in Table S5.

### Recombinant protein expression and enzyme assay

The full‐length coding sequences of *OsPDX1.2* and *OsPDX2* were amplified using Nipponbare (NIP) cDNA as a template and recombined into a modified pGEX‐6p‐1 vector (Invitrogen) containing a glutathione S‐transferase (GST) tag. The recombinant vectors were transformed into *E. coli* strain BL21 (DE3), and protein expression was induced by adding 0.1 mM isopropyl‐β‐D‐thiogalactoside (IPTG), followed by protein purification. The presence of target proteins was confirmed by sodium dodecyl sulfate polyacrylamide gel electrophoresis (SDS‐PAGE), and the purified recombinant proteins were selected for an enzyme activity assay. The primers used for these constructs are detailed in Table S5.

The *in vitro* enzymatic reaction of *OsPDX1.2* was conducted in a total volume of 100 μL reaction mixture, which contained 10 mM aldehydo‐D‐ribose 5‐phosphate (R5P), 50 mM D‐glyceraldehyde 3‐phosphate (G3P), 200 mM L‐glutamine (Gln) as substrates, 1 M Tris‐HCl buffer (pH 8.0), 100 mM MgCl_2_, and 5 μg of purified protein. The reaction was incubated at 37°C in the dark. After 30 min of incubation, 100 μL of ice‐cold methanol was added to terminate the reaction. The reaction mixture was filtered through a 0.22‐μm filter membrane (Anpel) prior to analysis by LC‐MS/MS. The content of pyridoxal‐5‐phosphate (PLP) was determined by peak integration calibrated with standard samples.

### Subcellular localization

To determine the subcellular localization of the OsPDX1.2 protein, the full‐length coding sequence of *OsPDX1.2* was amplified and the PCR product was cloned into pH7WGF2.0 (PJC032) to generate the 35S:EGFP–OsPDX1.2 construct. This construct, together with the plasma membrane marker CBL1‐OFP, was transiently expressed in *N. benthamiana* leaves via *Agrobacterium tumefaciens*‐mediated infiltration, and was also maxi‐prepared and transformed into protoplasts isolated from ZH11 rice seedlings. Two d after transformation (for tobacco) or after overnight incubation (for protoplasts), fluorescence signals were observed under a confocal laser scanning microscope (Zeiss, LSM980). The primers used for these constructs are detailed in Table S5.

### Transient dual‐luciferase reporter assay

To examine the activity of *OsPDX1.2* promoter haplotypes, the *OsPDX1.2* promoter sequences from NIP (*japonica*) and ZS97 (*indica*) were amplified and cloned into a modified pH2GW7 (PJG077) vector containing both firefly luciferase (LUC) and Renilla luciferase (REN) genes. These plasmids were introduced into *A. tumefaciens* strain *EHA105* and infiltrated into *N. benthamiana* leaves. Luciferase activities were measured using the Dual‐Luciferase Reporter Assay System (Promega) following the manufacturer's instructions.

For investigating the regulatory effect of OsbZIP18 on *OsPDX1.2*, site‐directed mutagenesis (vg1000082732, G‐C) was performed using the aforementioned amplified NIP promoter as a template, and the resulting sequence was ligated into the PJG077 vector. The full‐length coding sequence of *OsbZIP18* was cloned into the pEAQ HT‐DEST2 (PJF754) vector to serve as the effector. Measurements were conducted using the same method as described above, and luciferase activity was calculated as the ratio of LUC to REN (LUC/REN). The primers used for these constructs are detailed in Table S5.

### Yeast one‐hybrid

The promoter sequence of *OsPDX1.2* was amplified and ligated into the pHIS2 reporter vector. The full‐length cDNA sequence of *OsbZIP18* was amplified and fused with the pGADT7 vector. These fusion vectors were then co‐transformed into Y187 yeast cells (Clontech) and cultured on SD/–Leu–Trp medium for 3 d, followed by transfer to SD/–Leu–Trp–His medium for screening. The corresponding combination of empty pGADT7 with the pHIS2‐*OsPDX1.2* was co‐transformed as a negative control.

### Electrophoretic mobility shift assay

The full‐length cDNA of *OsbZIP18* was cloned into the pGEX‐6p‐1 vector with a GST tag. This recombinant plasmid was then transformed into *E. coli* strain BL21 (DE3) cells for protein expression, and the recombinant OsbZIP18 protein was obtained following purification. FAM‐labeled probes containing the ACE motif were synthesized for electrophoretic mobility shift assay (EMSA). Following this, reaction mixtures were prepared and incubated as previously described (Xian et al., 2025). The relevant primer and probe sequences are listed in Table S5.

### Exogenous application of VB6 and ABA

All seeds were germinated on distilled water‐saturated filter paper at 37°C for 3 d. After germination, the seeds were transferred to plastic containers for hydroponic culture in a climate chamber, with growth conditions maintained as follows: white light intensity of 120 μmol m^−2^ s^−1^, photoperiod of 16 h light/8 h dark, and temperature of 25°C. A dose–response analysis determined that 30 μM PN (VB6) and 30 μM ABA were the optimal concentrations for exogenous application. Twelve‐d‐old seedlings were subjected to cold treatment at 4°C for 5 d, followed by a 7‐d recovery period under 25°C conditions. For VB6 and ABA treatments, 30 μM solutions of pyridoxine and ABA were sprayed onto the leaves of rice seedlings prior to the initiation of cold treatment. Spray applications continued daily throughout the 5‐d cold treatment period, while control seedlings were sprayed with an equal amount of water. Plants were cultivated using a basal nutrient solution as described previously (Yoshida et al., 1976). The nutrient solution was renewed every 3 d.

### DAB and NBT staining

The diaminobenzidine (DAB; Sangon Biotech) and nitrotetrazolium blue chloride (NBT; Sangon Biotech) staining assays were performed following the method as previously described, with minor modifications (Liu et al., 2025b). Following a 24‐h treatment at 4°C, leaf samples were immediately collected and immersed in centrifuge tubes containing either 0.05% DAB (w/v) or 0.1% NBT (w/v) staining solution. The samples were then subjected to vacuum infiltration for 30 min and subsequently incubated in the respective staining solutions for 12 h (DAB) or 8 h (NBT) in the dark. Finally, the stained leaves were placed in 95% ethanol and incubated at 80°C until the decolorizing solution turned colorless.

### Quantification of malondialdehyde contents

The malondialdehyde (MDA) content in rice leaves was determined using the MDA content assay kit (D799761‐0050; Sangon Biotech). Approximately 0.1 g of leaf tissue was homogenized in 1 mL of ice‐cold extraction buffer. The homogenate was then centrifuged at 4°C and 8,000 *g* for 10 min. The supernatant was collected, and the MDA detection working solution was added to it. The absorbance values were measured at 532 nm and 600 nm, and these readings were used to calculate the MDA content.

### Statistical analysis

Data were analyzed using GraphPad Prism 8.0 and Microsoft Office Excel. Statistical significance between groups was determined by one‐way ANOVA (Tukey's test), the Wilcoxon rank‐sum test, and a two‐tailed Student's *t*‐test, as appropriate. Results are expressed as means ± *SE*, and exact *P*‐values are included in the figure legends.

### Accession numbers

Sequence data used in this study can be found in the Rice Genome Annotation Project (http://rice.plantbiology.msu.edu/) under the following accession numbers: *OsPDX1.2* (LOC_Os10g01080), *OsPDX2* (LOC_Os02g03740), and *OsbZIP18* (LOC_Os02g10860). The relative contents of pyridoxine (PN) and pyridoxine‑glucoside (PN‑G) for the 512 rice accessions are provided in Dataset S1.

## CONFLICTS OF INTEREST

The authors declare no conflicts of interest.

## AUTHOR CONTRIBUTIONS

Y.Z. and C.J. conceived and designed the study. B.W., Q.W., and W.S. performed the majority of experiments; R.L., X.Z., L.W., S.X., K.C., J.Z., Y.S., J.H., and X.L. assisted with experiments. Y.Z. and C.J. wrote and finalized the manuscript; A.F., Y.H., J.L., and J.L. discussed the results and revised the paper, and C.J. finalized the paper. All authors have read and approved the contents of this paper.

## Supporting information

Additional Supporting Information may be found online in the supporting information tab for this article: http://onlinelibrary.wiley.com/doi/10.1111/jipb.70322/suppinfo

**Dataset S1.** Relative contents of pyridoxine (PN) and pyridoxine‐glucoside (PN‐G) in 512 rice (*O. sativa* L.) varieties

**Dataset S2.** Rice accessions used for geographical distribution analysis of *OsPDX1.2* promoter haplotypes

**Figure S1.** Chromatographic profiles of vitamin B6 vitamers and their derivatives in the sample
**Figure S2.** Genetic composition and variation in vitamin B6 metabolite contents across 512 rice accessions
**Figure S3.** Dissection of a genetic locus underlying natural variations of vitamin B6 in rice
**Figure S4.** Subcellular localization of *OsPDX1.2*
**Figure S5.** Metabolic profiling of *ospdx1.2* mutants
**Figure S6.** Metabolic profiling of *OsPDX1.2*‐overexpressing plants
**Figure S7.** Expression analysis of *OsPDX1.2* under cold stress
**Figure S8.** Enhanced cold tolerance of *OsPDX1.2*‐overexpressing plants
**Figure S9.** ABA content in leaves of *ospdx1.2* mutants and *OsPDX1.2‐*overexpressing plants
**Figure S10.** Phenotypic analysis of cold tolerance in 36 *indica* accessions grouped by *pOsPDX1.2* haplotypes
**Figure S11.** Metabolic profiling of *osbzip18* mutant plants
**Figure S12.** Expression levels of ABA biosynthesis‐ and signaling‐related genes in the *ospdx1.2* mutant under cold stress
**Table S1.** Detailed information of the detected metabolites of 7 types of VB6 and their derivatives in this study
**Table S2.** Summary of candidate genes assigned from mGWAS results
**Table S3.** Information related to SNPs in *OsPDX1.2*
**Table S4.** List of bZIP transcription factors co‐expressed with *OsPDX1.2* identified through the ATTED‐II database
**Table S5.** Primers and probes used in this study
