# Supplementary material for: Natural variation in the promoter of OsPDX1.2 enhances vitamin B6 accumulation and cold tolerance in rice
Source: J Integr Plant Biol. 2026 Jun 15;68(11):3914–28. doi: 10.1111/jipb.70322 (PMC13725607; doi:10.1111/jipb.70322)
Supplement: Supplementary file 1 [file JIPB-68-3914-s001.docx]

**Figure S1. Chromatographic profiles of vitamin B6 vitamers and their derivatives in the sample**

Relative intensities of vitamin B6-related compounds are plotted against retention time (min). Corresponding chemical structures are annotated adjacent to their respective chromatographic peaks.

**Figure S2. Genetic composition and variation in vitamin B6 metabolite contents across 512 rice accessions**

**(A)** Pie chart showing the distribution of the 512 rice accessions. **(B–E)** Frequency distribution of the contents of pyridoxine **(B)**, pyridoxine-glucoside **(C)**, 4-pyridoxic acid **(D)**, and 4-pyridoxic acid-glucoside **(E)** in 512 rice (*O. sativa* L*.*) accessions. **(F–I)** Comparison of PN **(F)**, PN-G **(G)**, 4PA **(H)** and 4PA-G **(I)** contents between *indica*, *japonica*, and others rice subspecies. Violin plots show the distribution of four specific compounds across subpopulations. The width of each violin represents the probability density of the data. Smoothing intensity was set to medium. *P* values were calculated using the Kruskal-Wallis test followed by Dunn’s post hoc test .

**Figure S3. Dissection of a genetic locus underlying natural variation of vitamin B6 in rice**

**(A)** Local Manhattan plots of GWAS results of PN and PN-G in 512 rice accessions. The corresponding linkage disequilibrium (LD) heatmap of the candidate region (delineated by red dashed lines) presented below. The candidate interval encompassing the peak is marked with red vertical lines in the Manhattan plots. Red dots denote SNPs located within the promoter region of *OsPDX1.2*, and the corresponding SNPs are highlighted with red lines in the LD heatmap. **(****B–C)** Local transcriptome-wide association study (TWAS) plots for PN **(B)** and PN-G **(C)** across a 0.24 Mb region on chromosome 10. Red dots indicate association signals corresponding to *OsPDX1.2*. **(D–E)** Correlation analysis between *OsPDX1.2* expression and the contents of PN **(D)** and PN-G **(E)** across 274 rice varieties. The *r* values were derived from Pearson correlation analysis. **(F)** Haplotype analysis of the polymorphic promoter region of *OsPDX1.2* in 512 rice varieties. **(G–I)** Comparative analysis of PN **(G)**, PN-G **(H)**, and *OsPDX1.2* expression level **(I)** among four distinct haplotypes. *P* values were calculated using the Kruskal-Wallis test followed by Dunn’s post hoc test.

**Figure S4. Subcellular localization of OsPDX1.2**

Subcellular localization of OsPDX1.2 assessed using an eGFP-OsPDX1.2 fusion construct. GFP signals were visualized in rice protoplasts, showing localization in the cytoplasm. Scale bar = 5 μm.

**Figure S5. Metabolic profiling of *ospdx1.2* mutant plants**

**(A)** Mutations induced by the CRISPR-Cas9 system in the *ospdx1.2* mutant, showing the *ospdx1.2-1* (+A), *ospdx1.2-2* (**–**G), and *ospdx1.2-3* (**–**AA) frameshift alleles. **(B–F)** PLP **(B)**, PL **(C)**, PM **(D)**, PN **(E)** and PN-G **(F)** content in grains of wild-type and *ospdx1.2* mutant plants. Data are shown as the means ± *SE* (*n* = 3). Statistical significance was determined by One-way ANOVA (Tukey's test).

**Figure S6. Metabolic profiling of *OsPDX1.2*-overexpressing plants**

**(A)** Transcript levels of *OsPDX1.2* in *OsPDX1.2*-overexpressing plants. **(B–F)** PLP **(B)**, PL **(C)**, PM **(D)**, PN **(E)** and PN-G **(F)** content in leaves of wild-type and *OsPDX1.2*-overexpressing plants. Data are shown as the means ± *SE* (*n* = 3). Statistical significance was determined by One-way ANOVA (Tukey's test).

**Figure S7. Expression analysis of *OsPDX1.2* under cold stress**

Twelve-day-old wild-type seedlings were subjected to 4°C treatment for 0, 6, 12, 24, and 48 hours. Expression levels at 25°C (0 h) served as the control. Data are shown as the means ± *SE* (*n* = 3).

**Figure S8. Enhanced cold tolerance of *OsPDX1.2*-overexpressing plants**

**(A)** Phenotypic analysis of cold tolerance in wild-type (ZH11) and *OsPDX1.2*-overexpressing (*OsPDX1.2*_*OE*) plants. Twelve-day-old plants grown under normal conditions were subjected to cold treatment at 4°C for 8 days, followed by a 7-day recovery at 25°C. Scale bar, 5 cm. **(B)** MDA contents in leaves of twelve-day-old wild-type and *OsPDX1.2*_OE rice seedlings after 24-hour treatment at 4°C. **(C–D)** Fresh weight **(C)** and survival rate **(D)** of plants measured after recovery from 4°C treatment. **(E)** Histochemical analysis of reactive oxygen species (ROS) in leaves under cold stress. Hydrogen peroxide (H₂O₂) and superoxide anion (O₂⁻) accumulation were detected using diaminobenzidine (DAB) and nitroblue tetrazolium (NBT) staining, respectively. Seedlings were treated at 4°C for 24 hours prior to staining. Scale bar, 1 cm. In **(B)** and **(D)**, data are presented as means ± *SE* (*n* = 3). In **(C)**, data are presented as means ± *SE* (*n* = 16). Statistical significance was determined by One-way ANOVA (Tukey's test).

**Figure S9. ABA content in leaves of *ospdx1.2* mutants (A) and *OsPDX1.2*-overexpressing plants (B)**

Data are shown as the means ± *SE* (*n* = 3). Statistical significance was determined by One-way ANOVA (Tukey's test).

**Figure S10. Phenotypic analysis of cold tolerance in 36 *indica* accessions grouped by *pOsPDX1.2* haplotypes**

Eight-day-old seedlings from 18 *pOsPDX1.2^Hap1^* (left) and 18 *pOsPDX1.2^Hap2^* (right) accessions were treated at 4°C for 4 days and recovered at 25°C for 7 days. Scale bar, 15 cm.

**Figure S11. Metabolic profiling of *osbzip18* mutant plants**

**(A)** Transcript levels of *OsPDX1.2* in *osbzip18* mutant plants. **(B–G)** PLP **(B)**, PL **(C)**, PM **(D)**, PN **(E)**, PN-G **(F)**, and ABA **(G)** content in leaves of wild-type and *osbzip18* mutant plants. Data are shown as the means ± *SE* (*n* = 3). Statistical significance was determined by One-way ANOVA (Tukey's test).

**Figure S12. Expression levels of ABA biosynthesis- (A) and signaling-related genes (B–C) in the *ospdx1.2* mutant under cold stress**

Relative expression was measured by quantitative qRT-PCR and normalized to *Ubiquitin*. Data are shown as the means ± *SE* (*n* = 3). Statistical significance was determined by One-way ANOVA (Tukey's test).

**Table S1. Detailed information of the detected metabolites of 7 types of VB6 and their derivatives in this study**

| Compound | Molecular Weight  (theoretical) | Formula | Ionization model | Retention time | Precursor ions (Q1) | Product ions (Q3) | DP (volts) | CE (volts) | Identification |
| --- | --- | --- | --- | --- | --- | --- | --- | --- | --- |
| Pyridoxamine | 168.09 | C_8_H_12_N_2_O_2_ | [M+H]^+^ | 0.90 | 169.09 | 134.00 | 40 | 30 | standard |
| Pyridoxine | 169.07 | C_8_H_11_NO_3_ | [M+H]^+^ | 1.13 | 170.10 | 134.10 | 70 | 30 | standard |
| Pyridoxal | 167.06 | C_8_H_9_NO_3_ | [M+H]^+^ | 1.10 | 168.00 | 150.00 | 40 | 30 |  |
| Pyridoxal  5’-Phosphate | 247.10 | C_8_H_10_NO_6_P | [M+H]^+^ | 1.49 | 248.10 | 140.10 | 60 | 25 | standard |
| Pyridoxine  O-glucoside | 331.15 | C_14_H_21_NO_8_ | [M+H]^+^ | 1.11 | 332.15 | 152.10 | 40 | 65 |  |
| 4-Pyridoxic acid  O-hexoside | 345.10 | C_14_H_19_NO_9_ | [M+H]^+^ | 1.43 | 346.10 | 184.10 | 20 | 60 |  |
| 4-Pyridoxic acid | 183.05 | C_8_H_9_NO_4_ | [M–H]^-^ | 1.50 | 182.05 | 138.00 | -60 | -20 | standard |

**Table S2. Summary of candidate genes assigned from mGWAS results**

| Metabolite | Population | Associated  SNP | *P*-value | PVE | Candidate gene | Description | Gene source |
| --- | --- | --- | --- | --- | --- | --- | --- |
| Pyridoxine | All | sf0700127030 | 1.62E-10 | 0.07 | LOC_Os07g01020 | SOR/SNZ protein | *OsPDX1.1* |
| Pyridoxine | All | sf0700127030 | 1.62E-10 | 0.07 | LOC_Os07g01310 | Glutamate receptor |  |
| Pyridoxine | All | sf0900104138 | 6.37E-09 | 0.06 | LOC_Os09g01140 | Enhancer-Binding Protein | *OsGeBP11* |
| Pyridoxine | All | sf0900104138 | 6.37E-09 | 0.06 | LOC_Os09g01470 | Enhancer-Binding Protein | *OsGeBP12* |
| Pyridoxine | All | sf1000074974 | 2.80E-14 | 0.10 | LOC_Os10g01044 | Chloroplast inner membrane transporter | *OsTIC62* |
| Pyridoxine | All | sf1000074974 | 2.80E-14 | 0.10 | LOC_Os10g01080 | SOR/SNZ protein | *OsPDX1.2* |
| Pyridoxine | All | sf1000074974 | 2.80E-14 | 0.10 | LOC_Os10g01480 | Inositol 1,3,4-trisphosphate 5/6-kinase | *OsITPK1* |
| Pyridoxine | *Ind* | sf0700057635 | 6.99E-10 | 0.07 | LOC_Os07g01020 | SOR/SNZ protein | *OsPDX1.1* |
| Pyridoxine | *Ind* | sf0700057635 | 6.99E-10 | 0.07 | LOC_Os07g01310 | Glutamate receptor |  |
| Pyridoxine | *Ind* | sf0900047912 | 6.43E-10 | 0.07 | LOC_Os09g01140 | Enhancer-Binding Protein | *OsGeBP11* |
| Pyridoxine | *Ind* | sf0900047912 | 6.43E-10 | 0.07 | LOC_Os09g01470 | Enhancer-Binding Protein | *OsGeBP12* |
| Pyridoxine | *Ind* | sf1000054591 | 2.09E-12 | 0.09 | LOC_Os10g01044 | Chloroplast inner membrane transporter | *OsTIC62* |
| Pyridoxine | *Ind* | sf1000054591 | 2.09E-12 | 0.09 | LOC_Os10g01080 | SOR/SNZ protein | *OsPDX1.2* |
| Pyridoxine | *Ind* | sf1000054591 | 2.09E-12 | 0.09 | LOC_Os10g01480 | Inositol 1,3,4-trisphosphate 5/6-kinase | *OsITPK1* |
| PN-Glucoside | All | sf0700262781 | 7.75E-12 | 0.08 | LOC_Os07g01020 | SOR/SNZ protein | *OsPDX1.1* |
| PN-Glucoside | All | sf0700262781 | 7.75E-12 | 0.08 | LOC_Os07g01310 | Glutamate receptor |  |
| PN-Glucoside | All | sf0700262781 | 7.75E-12 | 0.08 | LOC_Os07g01750 | MATE efflux family protein |  |
| PN-Glucoside | All | sf0700262781 | 7.75E-12 | 0.08 | LOC_Os07g01760 | Glutamate:glyoxylate aminotransferase | *GGAT* |
| PN-Glucoside | All | sf0900043024 | 1.10E-10 | 0.08 | LOC_Os09g01140 | Enhancer-Binding Protein | *OsGeBP11* |
| PN-Glucoside | All | sf0900043024 | 1.10E-10 | 0.08 | LOC_Os09g01470 | Enhancer-Binding Protein | *OsGeBP12* |
| PN-Glucoside | All | sf1000072755 | 2.60E-14 | 0.10 | LOC_Os10g01044 | Chloroplast inner membrane transporter | *OsTIC62* |
| PN-Glucoside | All | sf1000072755 | 2.60E-14 | 0.10 | LOC_Os10g01080 | SOR/SNZ protein | *OsPDX1.2* |
| PN-Glucoside | All | sf1000072755 | 2.60E-14 | 0.10 | LOC_Os10g01480 | Inositol 1,3,4-trisphosphate 5/6-kinase | *OsITPK1* |
| PN-Glucoside | *Ind* | sf0700102970 | 2.84E-14 | 0.11 | LOC_Os07g01020 | SOR/SNZ protein | *OsPDX1.1* |
| PN-Glucoside | *Ind* | sf0700102970 | 2.84E-14 | 0.11 | LOC_Os07g01310 | Glutamate receptor |  |
| PN-Glucoside | *Ind* | sf0900043024 | 3.65E-09 | 0.07 | LOC_Os09g01140 | Enhancer-Binding Protein | *OsGeBP11* |
| PN-Glucoside | *Ind* | sf0900043024 | 3.65E-09 | 0.07 | LOC_Os09g01470 | Enhancer-Binding Protein | *OsGeBP12* |
| PN-Glucoside | *Ind* | sf1000079974 | 6.69E-11 | 0.08 | LOC_Os10g01044 | Chloroplast inner membrane transporter | *OsTIC62* |
| PN-Glucoside | *Ind* | sf1000079974 | 6.69E-11 | 0.08 | LOC_Os10g01080 | SOR/SNZ protein | *OsPDX1.2* |
| PN-Glucoside | *Ind* | sf1000079974 | 6.69E-11 | 0.08 | LOC_Os10g01480 | Inositol 1,3,4-trisphosphate 5/6-kinase | *OsITPK1* |

**Table S3. Information related to SNPs in *OsPDX1.2***

| SNP ID | Chromosome | Position | Distance to ATG  (bp) | Primary Allele | Secondary Allele | Effect |
| --- | --- | --- | --- | --- | --- | --- |
| vg1000082215 | chr10 | 82215 | –41 | G | A | UTR_5_PRIME |
| vg1000082230 | chr10 | 82230 | –56 | C | G | UTR_5_PRIME |
| vg1000082732 | chr10 | 82732 | –558 | G | C | promoter |
| vg1000082786 | chr10 | 82786 | –612 | C | G | promoter |
| vg1000082901 | chr10 | 82901 | –727 | G | A | promoter |
| vg1000082917 | chr10 | 82917 | –743 | C | T | promoter |
| vg1000083072 | chr10 | 83072 | –898 | G | T | promoter |

**Table S4. List of bZIP transcription factors co-expressed with *OsPDX1.2* identified through the ATTED-II database**

| Rank | Average coex z  to query genes | Locus ID | Function | Entrez Gene  ID |
| --- | --- | --- | --- | --- |
| 12 | 4.8 | LOC_Os02g10860 | Transcription factor HY5 | 4328662 |
| 410 | 1.8 | LOC_Os01g11350 | Basic leucine zipper 2-like | 4325565 |
| 604 | 1.6 | LOC_Os06g50310 | Basic leucine zipper 19 | 4342078 |
| 509 | 1.5 | LOC_Os01g07880 | Transcription factor HY5 | 4327123 |

**Table S5. Primers and probes used in this study**

| Primer name | Primer sequence (5'-3') | Purpose |
| --- | --- | --- |
| attB1 | AAAAAGCAGGCTTA | Gateway adaptor |
| attB2 | AGAAAGCTGGGTA | Gateway adaptor |
| OsPDX1.2-CDS-F | attB1-ATGGCCTCCGACGGCA | Gateway entry clone |
| OsPDX1.2-CDS-R | attB2-CTAGTCGGAGCGGGCGG | Gateway entry clone |
| OsPDX2-CDS-F | attB1-ATGGCAGTTGTTGGTGTACTC | Gateway entry clone |
| OsPDX2-CDS-R | attB2-TTATTCGAAGATTGGCAAGTC | Gateway entry clone |
| OsbZIP18-CDS-F | attB1-TGGTCCATGCTTCACGCAAG | Gateway entry clone |
| OsbZIP18-CDS-R | attB2-TCAATTCAAACCCAGAAGCATATTC | Gateway entry clone |
| OsPDX1.2-Pro-F | attB1-TGTTCTTTGATTTCCCGTTCT | Promoter_LUC |
| OsPDX1.2-Pro-R | attB2-GGGCGTCAGTGTTTGTGTT | Promoter_LUC |
| vg1000082732 site-directed mutagenesis-F | TAAGTTCACTTTACGTCCGCC | Promoter_LUC |
| vg1000082732 site-directed mutagenesis-R | GGCGGACGTAAAGTGAACTTA | Promoter_LUC |
| OsPDX1.2-qRT-F | ATCGTTGAGTTGTTATTGCCAC | qRT-PCR |
| OsPDX1.2-qRT-R | GAGAAGCTACCGGACTTGTG | qRT-PCR |
| OsbZIP18-qRT-F | GAACCAGATGCTCAGACAGATAC | qRT-PCR |
| OsbZIP18-qRT-R | CTTGCTGTCATCTTCTCCTCTATT | qRT-PCR |
| Ubiquitin-F | AACCAGCTGAGGCCCAAGA | qRT-PCR |
| Ubiquitin-R | ACGATTGATTTAACCAGTCCATGA | qRT-PCR |
| OsPDX1.2-F | AACGTTCAAATGGCGGACCTAAAGTGAACTTATTCCTGTG | EMSA |
| OsPDX1.2-R | CACAGGAATAAGTTCACTTTAGGTCCGCCATTTGAACGTT | EMSA |
